# A Systematic Review of Automatic Health Monitoring in Calves: Glimpsing the Future From Current Practice

**DOI:** 10.3389/fvets.2021.761468

**Published:** 2021-11-26

**Authors:** Dengsheng Sun, Laura Webb, P. P. J. van der Tol, Kees van Reenen

**Affiliations:** ^1^Farm Technology Group, Wageningen University and Research, Wageningen, Netherlands; ^2^Animal Production Systems Group, Wageningen University and Research, Wageningen, Netherlands; ^3^Livestock Research, Research Centre, Wageningen University and Research, Wageningen, Netherlands

**Keywords:** calf, early disease detection, precision livestock farming, sensor, health monitoring

## Abstract

Infectious diseases, particularly bovine respiratory disease (BRD) and neonatal calf diarrhea (NCD), are prevalent in calves. Efficient health-monitoring tools to identify such diseases on time are lacking. Common practice (i.e., health checks) often identifies sick calves at a late stage of disease or not at all. Sensor technology enables the automatic and continuous monitoring of calf physiology or behavior, potentially offering timely and precise detection of sick calves. A systematic overview of automated disease detection in calves is still lacking. The objectives of this literature review were hence: to investigate previously applied sensor validation methods used in the context of calf health, to identify sensors used on calves, the parameters these sensors monitor, and the statistical tools applied to identify diseases, to explore potential research gaps and to point to future research opportunities. To achieve these objectives, systematic literature searches were conducted. We defined four stages in the development of health-monitoring systems: (1) sensor technique, (2) data interpretation, (3) information integration, and (4) decision support. Fifty-four articles were included (stage one: 26; stage two: 19; stage three: 9; and stage four: 0). Common parameters that assess the performance of these systems are sensitivity, specificity, accuracy, precision, and negative predictive value. Gold standards that typically assess these parameters include manual measurement and manual health-assessment protocols. At stage one, automatic feeding stations, accelerometers, infrared thermography cameras, microphones, and 3-D cameras are accurate in screening behavior and physiology in calves. At stage two, changes in feeding behaviors, lying, activity, or body temperature corresponded to changes in health status, and point to health issues earlier than manual health checks. At stage three, accelerometers, thermometers, and automatic feeding stations have been integrated into one system that was shown to be able to successfully detect diseases in calves, including BRD and NCD. We discuss these findings, look into potentials at stage four, and touch upon the topic of resilience, whereby health-monitoring system might be used to detect low resilience (i.e., prone to disease but clinically healthy calves), promoting further improvements in calf health and welfare.

## Introduction

Diseases, in particular bovine respiratory disease (BRD) and neonatal calf diarrhea (NCD), are the most common causes of morbidity and mortality in veal calves (1), dairy calves (2), and beef youngstock (3). Despite slightly different prevalence rates (4), disease types affecting dairy and veal calves are similar (5–7). BRD symptoms include hampered respiration, nasal discharge, and coughing (5). A direct symptom of NCD is extremely watery feces (8). Potential risk factors for BRD include: inadequate passive transfer of immunity from colostrum (2, 9); low body weight at arrival in veal calves (10); poor indoor housing conditions compared to outdoor housing (10); and management practices such as weaning, comingling, and castration (11). Potential risk factors for NCD include: high exposure to pathogens causing NCD; factors related to host resistance or susceptibility to disease, e.g., low quality and quantity of colostrum; and factors about the environment that favor the host or agent, e.g., high stocking density and too high or too low ambient temperature and air humidity (12, 13).

Diseases in calves cause significant economic losses (14, 15), due to treatment (16), impaired growth and mortality (17). Diseases also impaired calf welfare (18). Moreover, antibiotic resistance, a major concern in human and veterinary medicine (19), is a serious problem in the veal (20, 21) and dairy industry (22). In addition, the overuse of antibiotics might result in the contamination of surface water near farms due to residues in the urine and feces of animals (23). Given the all-encompassing impact of calf health on sustainability aspects, it is essential that we develop accurate, timely, and practical systems to identify sick calves, in both the dairy and veal sectors.

The common practice for identifying diseases in calves is based on visual appraisal and clinical examinations performed by farmers and veterinarians (5). This practice is linked to a number of disadvantages: (1) Calves identified as sick already show clear clinical symptoms and may have already been sick for a while. For example, clinical signs of BRD might occur later than onset of fever (24), or even without the occurrence of fever (5), and clinical signs of NCD are visible when much of the associated tissue damage to the intestinal submucosa has already occurred (25). (2) Visual appraisal and clinical examinations are typically poor at identifying sick calves. For example, in a study diagnosing BRD in beef calves using clinical examination, the estimated sensitivity and specificity were 61.8 and 62.8%, respectively (26). Many sick calves, hence, go undetected, or require re-treatment due to delayed intervention and inappropriate antimicrobial dosage for the first case, which makes it difficult to promptly treat them, leading to greater chances of spread of disease, poorer animal welfare, and greater negative impacts on economy and environment, overall leading to poor sustainability of production systems involving calves.

Improved methods to detect health problems accurately and on a timely basis in individual calves are warranted. The decreasing cost and increasing implementation of electronic tools allows for the application of “sensing solutions” to animal farming. Behavioral and physiological parameters can nowadays be automatically recorded at individual animal level, continuously and over long periods of time (27, 28). During the past decade, various sensor data models have been proposed for automatic health-monitoring systems in dairy and veal calves. To date, however, there has been no systematic review presenting the associated gaps in research, while literature reviews have previously been done for pigs (29, 30), dairy cows (31, 32), dairy sheep (33), and dairy calves (27, 28). The objectives of this literature review were hence: to investigate previously applied sensor validation methods and gold standards; to identify sensors used on calves, the parameters these sensors monitor, and the statistical tools applied to identify diseases; and to explore potential research gaps to point to opportunities for future research.

## Methods

### Definitions

Animals included in this review were bovine animals aged <1 year; these include “calf” or “calves” (pre-weaned or weaned), heifers (weaning to 1 year of age), growing bulls (after arrival at the fattening farm up to 1 year of age), and beef cattle (early fattening period until 1 year of age). Precision livestock farming (PLF) is defined based on Berckmans (34) as “measuring variables on the animals, modeling these data to select information, and then using these models in real time for monitoring and control purposes”. We defined the following terms–SENSOR: an automatic tool capable of recording activities, behaviors, physiology, and body size of calves continuously; MODEL: a mathematical tool that describes the relations between the sensor output and the actual values of the measured parameters of the physical environment; VALIDATION: the process of determining the measurement ability of automatic tools relative to a gold standard using statistics. DISEASE: sickness status of an animal occurred naturally or induced by disease challenges.

**Figure 1:**
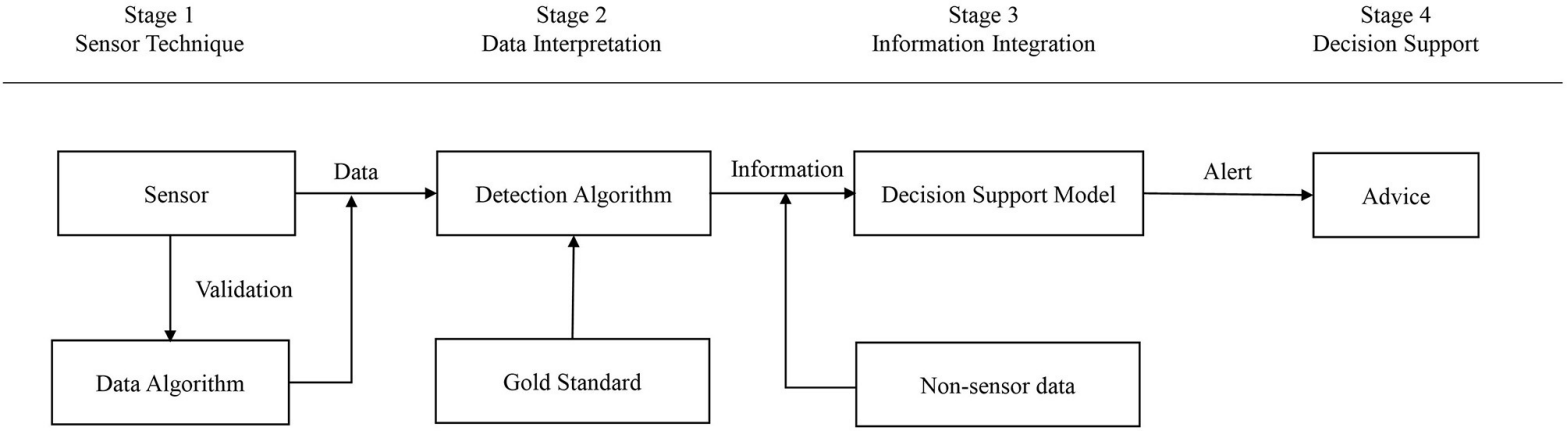
Four-stage development approach.

We defined four stages of development of a particular sensor technique for disease detection based on Rutten et al. (31) (Figure 1):

- *Stage one*: SENSOR TECHNIQUE-applying sensor technology to automatically or manually record behavioral or physiological parameters in animals, visualizing these parameters.
- *Stage two*: DATA INTERPRETATION-changes in data are detected and connected to changes in behavior and physiology with an established link to the animal's health status;
- *Stage three*: INFORMATION INTEGRATION-multiple data resources, e.g., treatment records and sensor data, are integrated to direct the farmer to potential problems that need attention;
- *Stage four*: DECISION SUPPORT–a sensor system that aids to make a decision, e.g., whether to treat an animal or not; what to treat the animal for?

### Inclusion and Exclusion Criteria

Peer-reviewed scientific articles describing applying sensors to calves were eligible for inclusion. Only articles based upon original data were included. Included articles were written in English, with complete, full-text documents available. To provide up-to-date review, only articles published between 2009 and 2021 were included. Manuscripts published after the completion of the literature search were not included (i.e., after May 10, 2021). Exclusion and inclusion criteria for the systematic review were based on an previous work by Beaver et al. (35) and agreed upon by all co-authors.

#### Search Strategy

Systematic searches were conducted using the Web of Science Core Collection database because it has high coverage rates of animal behavior and welfare and bio-system engineering journals with significant PLF contents.

The following search terms were applied: (calf OR calves OR dairy calf OR dairy heifer OR heifer calf OR heifers OR young cattle) AND (BRD OR bovine respiratory disease OR calf comfort OR calf health OR diarrhea OR group housing OR health OR precision livestock OR precision livestock farming OR proneness to disease OR welfare) AND (automatic OR automated measurement OR automated measures OR detection OR diagnosis OR disease monitoring OR evaluation OR modeling OR non-invasive detection OR prediction OR validation) AND (accelerometer OR activity sensor OR artificial intelligence OR automatic milk feeder OR bioacoustics OR computer vision OR electronic monitoring OR infrared thermography OR low-cost sensor OR non-invasive technology OR radio frequency identification OR reticulo-rumen bolus OR statistical process control OR sound analysis OR 3-D sensor). The selection of these search terms was based on initial screening of relevant articles to gain general background information and expert opinion.

#### Selection Process

**Figure 2:**
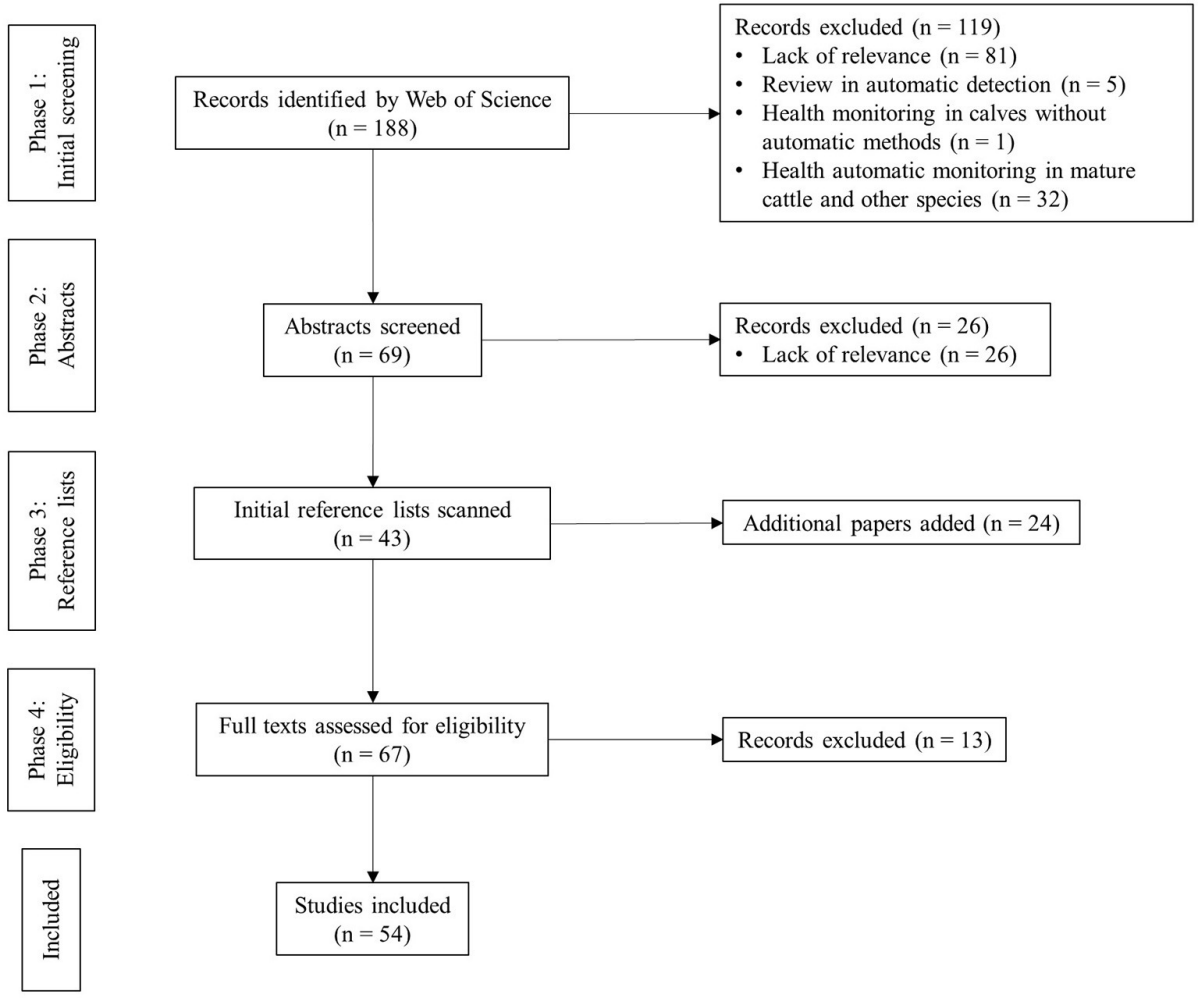
Article selection process.

The primary outcomes were selected based upon a four-step screening and appraisal process (Figure 2):

- Step one. Scanning the titles–filter out irrelevant results such as review articles in automatic detection, original articles of health monitoring in calves without applying sensor technology, or original articles of automatic health-monitoring systems in mature cattle or other species.
- Step two. Evaluating abstracts–identify and remove irrelevant articles.
- Step three. Snowballing–checking and selecting references within selected articles.
- Step four. Eligibility. Full texts of the remaining articles were read in detail. Original experimental studies were excluded if not aiming at health monitoring in calves aged up to 1 year using sensor technology.

#### Data Extraction

From each included article, where applicable, we recorded the objectives, animal category, parameters measured, sample size, gold standards for validation, sensors used, and measurements used to assess the performance of the sensors or algorithms. Missing information was noted down as “not available.” The results were pooled in the form of a table (Supplementary Material). The reliability for data extraction was tested by author 1 (DS) on a random subset of 20 articles, with a result of 100% agreement.

### Data Management

Extracted data were entered into and managed in excel spreadsheets (version 2016, Microsoft Corp., Redmond, WA, RRID:SCR_016137).

## Results and Discussion

**Figure 3:**
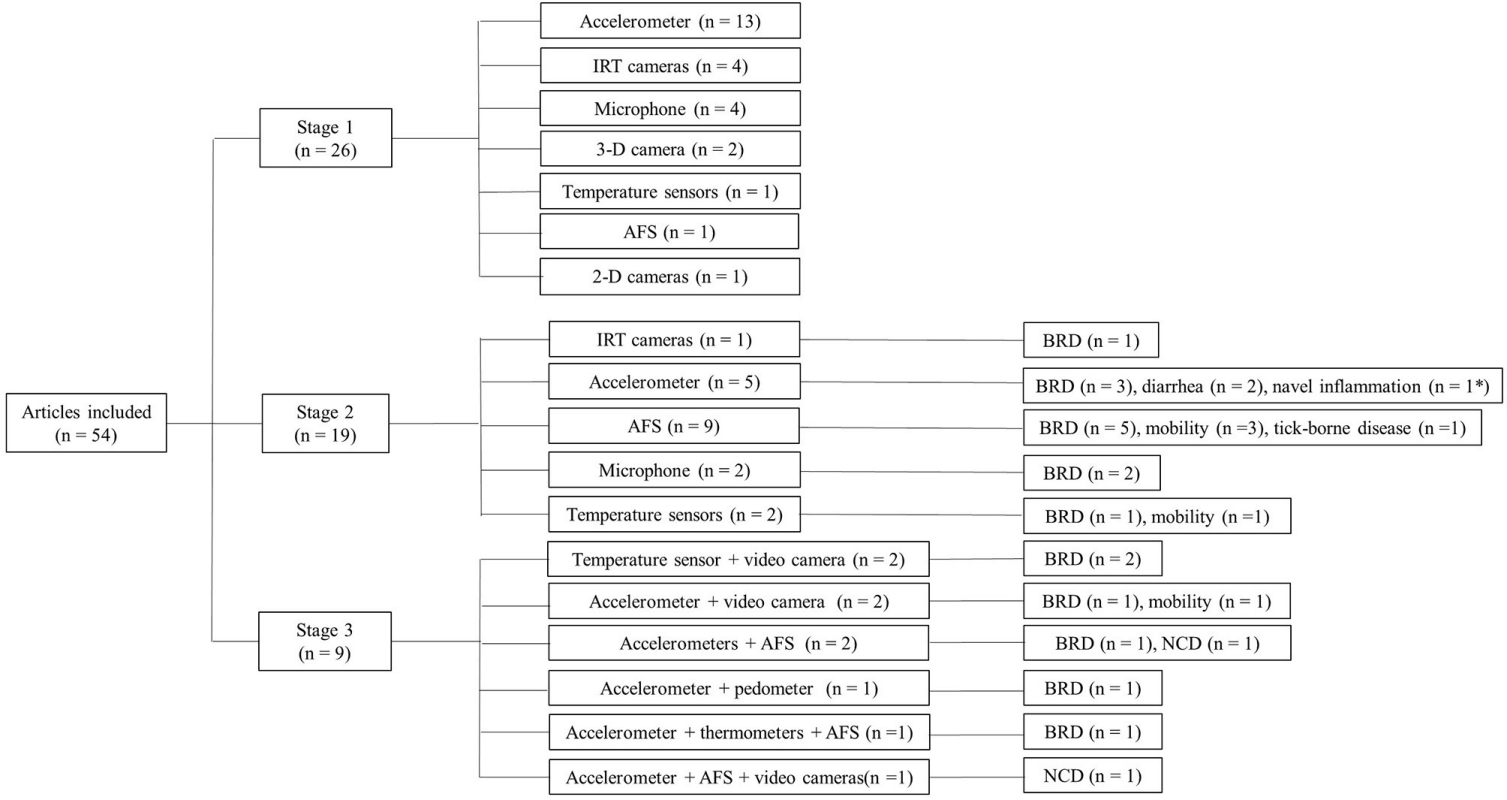
Distribution of stages of included articles. *In Studds et al. (56) both diarrhea and navel inflammation were studied.

Following the article-selection process described above, 54 articles were included in this review (Figure 2). As shown in Figure 3, 26 articles fell into *stage one* (sensor technique), 19 articles fell into *stage two* (data interpretation), and 9 articles fell into *stage three* (information integration). We found no articles at *stage four* (decision support).

Studies at these different stages use different validation methods and gold standards. Studies at stage one aim to check that a given sensor is accurately recording a particular behavioral or physiological parameter of interest. These studies typically use (a) manually collected parameter(s) as gold standard for their validation, e.g., video observations of lying bouts or rectal body temperature measurements using a thermometer. Stage two and stage three studies aim to identify sick calves as early as possible. Stage two and three studies develop and test algorithms applied to sensor data to accurately detect sick individuals. Manual health-assessment protocols are typically used as gold standards to develop and test these algorithms (Table 1).

**Table 1:** Gold standards of studies at stage two and three.

**No**.	**Reference**	**Stage**	**Gold standard**		
			**Clinical examination**	**Blood analysis**	**Other**
1	Borderas et al. (45)	2	Yes (daily)		
2	Timsit et al. (24)	2	Yes (twice daily)	Yes	
3	Schaefer et al. (16)	2	Yes (daily)	Yes	
4	Moya et al. (58)	2	Yes (frequency information not available)		Carcass information, lung lesions
5	Wolfger et al. (61)	2	Yes (twice daily)	Yes	
6	Jackson et al. (55)	2	Yes (at least twice daily)		BW
7	Johnston et al. (52)	2	Yes (modified Wisconsin calf health scoring chart: twice weekly in pre-weaning and weaning periods and once weekly in post-weaning period)	Yes	
8	Pillen et al. (81)	2	Yes (daily)		Depression score
9	Vandermeulen et al. (51)	2	Yes (Wisconsin calf clinical respiratory score: at least twice weekly in pre-weaning period and once weekly in post-weaning period)	Yes	
10	Voss et al. (46)	2	Yes (at least twice daily)		
11	Knauer et al. (53)	2	Yes (Wisconsin calf clinical respiratory score: daily)	Yes	Calf enrollment, treatment record, morbidity and mortality data
12	Swartz et al. (47)	2	Yes (Wisconsin calf health scoring chart: twice daily)		
13	Carpentier et al. (65)	2	No examination	Yes	
14	Knauer et al. (54)	2	Yes (Wisconsin calf clinical respiratory score: daily)		Calf enrollment, treatment record, morbidity and mortality data
15	Oliveira et al. (62)	2	Yes (daily)	Yes	
16	Shane et al. (49)	2	Yes (daily)		
17	Studds et al. (56)	2	Yes (twice weekly)		
18	Kayser et al. (57)	2	Yes (twice weekly)		BW
19	Swartz et al. (63)	2	Yes (Wisconsin calf health scoring chart: twice weekly)		
20	Hanzlicek et al. (60)	3	Yes (three times daily)	Yes	
21	Szyszka et al. (66)	3	Rectal temperature (day 0, 13, 15, 17, 20, 27, and 31); fecal samples (day 0, 13, 15, 17, 20, 27)	Yes	BW
22	Toaff-Rosenstein et al. (59)	3	Yes (daily)		Necropsy
23	Toaff-Rosenstein and Tucker (50)	3	Yes (daily)		
24	Hixson et al. (48)	3	Yes (Wisconsin calf health scoring chart: twice daily)		
25	Sutherland et al. (4)	3	Yes (daily)	Yes	BW
26	Lowe et al. (7)	3	Yes (daily)		
27	Kayser et al. (72)	3	Yes (twice daily)	Yes	
28	Duthie et al. (64)	3	Yes (modified Wisconsin calf health scoring chart: daily)		

We first define what is meant by “validation” in this review as well as define the terms used in this context, i.e., sensitivity, specificity, accuracy, and positive and negative predictive value. We follow up with a description of the different gold standards that have been used at the different stages of investigation. Next, we describe the various sensors that have been used in calf-health-monitoring research, the parameters these sensors record, and their accuracies in these recordings. We end by presenting the current research at stage two (data interpretation) and stage three (information integration), revealing important knowledge gaps between stage three and stage four (decision support), suggesting the direction for future study that will enable the bridging of these gaps, hence reaching automated health-related data interpretation and complete decision-support systems for calf production systems.

### Validation

The validation assessments at different stages of studies share common principles. Validation assessments are typically calculated via so-called confusion matrices (Table 2) (36). Confusion matrices reveal relationships between the sensor of interest, the selected gold standard (see the below formulas for: sensitivity, specificity, accuracy, precision, and negative predictive values) and the underlying prevalence of the disease interest. “Positive and negative” show the sensor (or model) output (a response of “yes” or “no” to the disease detection), while “true and false” reflects whether the sensor (or model) output is in line with the gold standards in a pre-specified time window (i.e., whether the prediction matches the reality). When comparing article outcomes, it should be noted that sensitivity and specificity are affected by characteristics of the sensor, while accuracy, precision, and negative predictive values are affected by the prevalence of disease or behavior based on the dataset: the higher the prevalence, the better the accuracy, precision, and predictive values for the given dataset. Model developments are usually aimed to enhance the contrast in a sensor system output for the purpose of threshold evaluations (e.g., sensitivity, specificity, or accuracy) over a given range. Common methods used for model developments are correlation, area under curve (36), and receiver operating characteristic curves (36).


$$\begin{array}{l} Sensitivity=\frac{true\;positives}{\left(true\;positives+false\;negatives\right)}\\ Specificity\;=\;\frac{true\;negatives}{\left(false\;positives\;+\;true\;negatives\right)}\\ Accuracy\\ =\;\frac{\left(true\;positives\;+\;true\;negatives\right)}{(true\;positives+\;true\;negatives\;+false\;positives}\\ \;+\;false\;negatives)\\ \;Precision\;(positive\;predictive\;value)\;\\ \;=\;\frac{true\;positives}{\left(true\;positives\;+\;false\;positives\right)}\\ Negative\;predictive\;value\;=\;\frac{true\;negative}{\left(true\;negative\;+\;false\;negative\right)} \end{array}$$

**Table 2:** Confusion matrix.

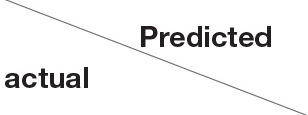	**Positive**	**Negative**
Positive	True positive	False negative
Negative	False positive	True negative

### Gold Standard

To obtain a sound validation of a sensor or PLF system, an objective “gold standard” is needed. In this regard, studies at stage one to stage four require different gold standards. At stage one, gold standard means “variables of interest,” i.e., behavioral or physiological parameters; at stage two, three, and four, gold standard usually refers to the identification of disease, typically via a manual “clinical examination.”

Stage one studies, where sensors are checked directly for their ability to record behavioral or physiological parameters, tend to use manual sampling of these behavioral or physiological parameters. For sensors recording behavioral parameters, behavioral observations of videos, continuous or at regular intervals, are a commonly used reference for validation. Continuous sampling of focal animals will provide the most accurate data for calf behavior, but is a time-consuming exercise. For certain, long-term, so-called “state” behaviors, instantaneous scan sampling at regular intervals may provide an accurate enough gold standard and is less time consuming, for example, meal time and frequency over a 3 day period can be detected accurately with instantaneous scan sampling at short intervals of 30 s and 1 min (37). However, one main disadvantage of video observation is that it is labor intensive, and requires training to achieve appropriate observer reliability; and observer error might occur (38).

For sensors recording physiological parameters, manual measurements of these physiological parameters are also used as gold standards. Sensors recording body temperature, e.g., body surface temperature (39), eye temperature (40, 41), and rectal area temperature (40), typically use manually recorded rectal temperature as gold standard. When validating body dimensions in calves and heifers, manual measurements of body weight and dimensions are common, including body weight (42, 43), hip height (42), and wither height (42).

With increasing research into validating sensors in terms of how accurately they record behavior or physiological parameters, previously validated sensors may be used as automated gold standard to validate new sensors, which significantly reduces labor required for these types of stage one studies. The Hobo Pedant G Data Logger, for example, has been previously used as a gold standard to validate another accelerometer, the AfiTag II, for lying behavior and step count (44).

Stage two and three studies aim to identify sick calves. Here, a clinical examination is the most commonly used gold standard for disease diagnosis (Table 1) (45–50). Various protocols have been used in this type of study, such as the Wisconsin clinical respiratory score (51) and the Wisconsin calf health scoring chart (52). Further information can be added to these clinical examinations to complement the gold standard, including metadata such as management information (e.g., calf registration or enrollment data), morbidity and mortality data from the farm (53, 54), BW (55–57), post-mortem examination (58, 59), or blood parameters (16, 24, 51–53, 60–62). Of all the clinical examination protocols, the (modified) Wisconsin calf health score chart was the most commonly used protocol (47, 48, 52, 63, 64). Gold standards without clinical examination, e.g., from blood analysis (65), or a combination of BW, biochemical parameters from blood and fecal samples, and rectal temperature (66) have also previously been used. Clinical examination can be combined with clinical chemistry, for example, via blood sampling, to improve the accuracy of health assessment.

Visual appraisal of disease, e.g., BRD, relies on the experience of observers, and may have low specificity and be highly variable between observers based on their level of experience (67). Thus errors from the clinical examinations may transfer to the corresponding models (58). Improvements are necessary for the clinical examinations used as gold standards for the development of algorithms to detect diseases in calves. Firstly, training in clinical examination and high inter-observer consistency are required. Secondly, to better relate clinical examinations to model outcome, consistent and explicit definitions of diseases across the literature are needed.

When performing time-consuming clinical examinations for use as a gold standard, the frequency of these examinations needs to be carefully considered. As observed by the current authors, daily clinical examinations of calves can provide better timely reference, at the cost of disturbance to the group and high labor requirements. However, a low frequency of clinical examinations will result in late detection, making it difficult to develop an early disease detection algorithm (68). Previous research applied different frequencies–for clinical examinations–ranging from daily to weekly (Table 1). Clinical examinations combining two different frequencies applied at different life stages were also found, e.g., before (twice a week) and after the weaning period (once per week) of dairy calves (51, 52). To the author's knowledge, no study has yet compared the effect of different frequencies of clinical examinations on the accuracy of disease-detection models.

In summary, clinical examination is the most common gold standard used in the development of algorithms to identify sick calves. The Wisconsin calf health score chart was identified as a commonly used protocol for clinical examination in this context, with a sensitivity of 62.4, and specificity of 74.1% (69). Clinical references with high accuracy, consistent guidelines, and easy-to-follow protocols are needed for disease detection in calves. A standardized clinical scoring system will benefit the validation of the sensors and algorithms, making it easier to compare the performance of different algorithms. In addition, ultrasonographic assessment of the thorax could be a useful tool to assess BRD detection in calves (70, 71).

### Stage One: Sensor Technology Used in Calves

Data sources used in calves include automatic feeding stations (AFS), accelerometers, microphones, infrared thermography (IRT) cameras, temperature sensors (i.e., boluses, thermometers), radiofrequency identification (RFID) chips, 3-D cameras, and 2-D cameras.

#### Automatic Feeding Stations

AFS, such as automated milk dispensers for pre-weaned calves and automatic concentrate bunks for post-weaned calves, and water bins, have been used in studies aimed at automated health monitoring in young calves, hence stage two research. These AFS can measure a wide range of parameters linked to feeding and drinking patterns, including daily feed intake (4, 45, 47, 52–55, 57, 72–74), frequency and duration of rewarded and unrewarded visits (4, 45, 47, 52–55, 57, 58, 61, 62, 72, 74), drinking speed (milk) (47, 52–54), water-drinking behavior (intake, time, and frequency) (62, 74), and other feeding behaviors (head-down duration at the AFS) (55, 72), time-to-bunk: time to approach feeding stations following feed-truck delivery (55, 72), and duration of unrewarded visit intervals (55). We did not find validation studies for common systems such as *Förster-Technik GmbH* (*n* = 5; Engen, Germany) or GrowSafe Systems (*n* = 3; GrowSafe Systems Ltd., Airdrie, AB, Canada). However, we found a study validating automatic feed and water bin (Intergado ® Ltd, Contagem, Minas Gerais, Brazil), and showed that this system seem to be able to measure feeding time, water drinking time, feed intake per visits, and water intake per visits with high correlation compared with the gold standards (*r*^2^ = 0.917, 0.963, 0.973, and 0.986, respectively) (74).

#### Accelerometers

Accelerometers are attached to the body of the calf, generally to one of the limbs, neck, or ear (tag). They are typically used to assess various activity-related behaviors. Accelerometers are accurate in recording calf behaviors, including lying time (44, 75–78), lying bouts (44, 75, 76, 78), standing time (75, 78), standing bouts (75), step counts (44, 79), locomotion time (78), gait scoring (79), feeding time (73, 78), sucking behavior from dams (38), and licking or sucking at objects, other calves' bodies, or own body (78). After more than 10 years of development, accelerometers are now used to record a broader variety of behaviors and more detailed behavioral patterns, e.g., recognizing between galloping, trotting, and walking (79), and recording behaviors such as eating, water drinking, chewing, positive social interactions, self-grooming, and inactivity (80). Step counts were originally measured by pedometers (60). This activity parameter was later integrated into accelerometers (44, 47, 66, 79, 81).

#### Temperature Sensors

Boluses, IRT cameras, and thermometers are used to measure body temperature. These temperature sensors have been developed to record body temperature at different anatomical areas, enabling the measurement of rectal temperature (50, 59) or temperature around the rectal area (40), reticulo-rumen temperature (24, 46), eye temperature (7, 16, 40, 41, 82), cheek temperature (7, 82), back, shoulder, and side temperature (7), and temperature at the base of the tail (39). These cameras have also shown high accuracy in measuring cheek temperatures (82), but have not been found to be highly accurate in measuring temperature around the rectal area (40) or core body temperature (41). In terms of eye temperature, IRT cameras seem to show varying levels of correlation between eye temperature and rectal temperature, e.g., high correlation (*R*^2^ ≥ 0.99) (82), low correlation (*R*^2^ ≤ 0.32) (40). This might be partly due to the use of an detection algorithm (82). A prototype thermometer provided by Nogami et al. (39) has been found to measure tail temperature with high correlation compared with rectal temperature in calves.

#### Other Sensors and Techniques

Microphones, when integrated into sound-acquisition systems, can detect abnormal cough sounds (51, 65, 83) and rumination sounds in calves (84–86). The performance of microphones varied in calves of different ages. Microphones accurately recorded rumination time in pre-weaned calves (85), but overestimated rumination time in weaning calves (86). RFID ear tags can be applied to monitor grooming behavior (measured via proximity to a brush) in heifers (87). IRT cameras have also been used to assess respiration rate in calves, at a high level of accuracy (88).

With the application of approaches such as computer vision or machine learning, an even broader range of parameters might be recorded with the available sensors. For example, Carslake et al. (89) applied machine-learning approaches to multi-class behavior identification (including locomotor play, self-grooming, ruminating, non-nutritive suckling, nutritive suckling, active lying, and non-active lying) as well as behavior quantification (i.e., behavior distribution) using a single sensor (comprised of an accelerometer and gyroscope) in calves. Computer vision allowed 2-D cameras to identify multiple behaviors, e.g., pen entering, pen leaving, standing or lying static behavior, turning, and feeding and drinking behaviors (90). 3-D cameras can monitor growth and morphology (i.e., BW, body mass, hip height, and wither height) in young calves and heifers (42, 43).

Knowing which parameters that sensors (or sensor combinations) can accurately measure can contribute to the development of an efficient sensor system at stage two and three. For example, accelerometers are not accurate in screening rumination time in calves (73), but this can instead be achieved by microphones (84, 85). Both accelerometers and AFS can record feeding and water-drinking behaviors, but AFS can record these behaviors directly without having to apply statistical models and are non-intrusive, i.e., not attached to the animal (80). In addition, no further hardware is needed when extracting data from AFS compared with accelerometers.

To sum up, available sensors (AFS, accelerometers, IRT cameras, microphones, and 3-D cameras) are accurate in measuring different behavioral or physiological parameters in calves, and approaches such as machine learning and computer vision broaden the range of behaviors that sensors can record. Future work should further develop behavior classification and quantification by applying computer-vision and machine-learning approaches.

### Stage Two: Data Interpretation–Outcomes of Algorithms

In order to develop a sensor-based system that detects sick calves, i.e., sensor technology combined with algorithms, stage two studies must follow three steps: (1) identify how behavioral or physiological parameters change with disease, identified via a gold standard (this includes the selection of both parameters of interest and corresponding sensors); (2) investigating how these behavioral and physiological changes vary at which stage of disease they can first be detected; and (3) developing and testing the accuracy (or performance) of algorithms in detecting sick calves based on changes in these behavioral and physiological parameters. In this section, we highlight the algorithms that can detect diseases prior to clinical confirmation (Table 3), and summarize changes in behavioral and physiological parameters in response to disease as well as time course: disease states in animals typically lead to both behavioral and physiological changes over time.

**Table 3:** Performance of algorithms and models.

**No**.	**References**	**Features**	**Performance**							
			**Se**^**a**^ **(%)**	**Sp**^**b**^ **(%)**	**Accuracy (%)**	**PPV**^**c**^ **(%)**	**NPV**^**d**^ **(%)**	**Other parameters**	**Days prior (best)**	**Days prior (least)**
1	Jackson et al. (55)	Feeding behavior							−14.2	−1.3
2	Kayser et al. (57)-univariate factors	Feeding behavior			48.7–80.1				−10.2	−0.6
3	Wolfger et al. (61)	Feeding behavior							−7	
4	Lowe et al. (7)	Feeding behavior, lying behavior, body temperature							−7	−4
5	Swartz et al. (63)	Activity, lying behavior							−7	−6
6	Jackson et al. (55)	DMI							−6.8	
7	24	Reticulo-rumen temperature						0.91 (r), 0.82 (r^2^)	−5.7	−0.5
8	Sutherland et al. (4)	Feeding behavior, lying behavior							−5	0
9	Pillen et al. (81)	Activity							−5	−1
10	Kayser et al. (72)	Feeding behavior			0.61–0.89				−4.5	
11	Kayser et al. (72)	Rumen temperature			0.78				−4.5	
12	Sutherland et al. (4)	Feeding behavior							−4	0
13	Knauer et al. (53)	Feeding behavior, activity							−4	7
14	Voss et al. (46)	Reticulo-ruminal temperature	71	98		86	98	0.855 (area under curve)	−3.5	
15	Moya et al. (58)-model 33	Feeding behavior	66.7	58.3	62.5				−3.1	
16	Moya et al. (58)-model 66	Feeding behavior	75	50	50				−3.1	
17	Knauer et al. (54)	Feeding behavior	56.4	49.5		66.6	49.5		−3.1	
18	Knauer et al. (54)	Feeding behavior	70.9	32.9		65.3	38.7		−3.1	
19	Knauer et al. (54)	Feeding behavior	74.9	27.1		64.6	37.4		−3.1	
20	Johnston et al. (52)	Feeding behavior							−3	
21	Oliveira et al. (62)	Feeding behavior							−3	4
22	Duthie et al. (64)	Feeding behavior, activity							−3	−1
23	Moya et al. (58)-model 14	Feeding behavior	58.3	83.3	70.8				−2.4	
24	Kayser et al. (57)-multivariate factors	Feeding behavior			84				−2.1	−2
25	Shane et al. (49)	Social network patterns			17.9–100	<10	>90		−2	0
26	Swartz et al. (47)	Behavior, activity							−2	
27	Sutherland et al. (4)	Feeding behavior							−2	0
28	Sutherland et al. (4)	Lying behavior							−2	0
29	Toaff-Rosenstein and Tucker (50)	Rectal temperature							−2	
30	Moya et al. (58)-model 3	Feeding behavior	50	100	75				−1	
31	Oliveira et al. (62)	Feeding behavior							−1	1
32	Toaff-Rosenstein and Tucker (50)	Feeding behavior							0	

a *refer to sensitivity.*

b *refer to specificity.*

c *refer to positive predictive value.*

d *refer to negative predictive value*.

#### Changes in Feeding Behaviors

Feeding behaviors and patterns, including intake, frequency, speed, and duration at various time ranges, are commonly used parameters for the early detection of disease in calves (91, 92). Note that most studies look at feeding behaviors aggregated at a daily level. With the application of RFID, individual calves are identified at AFS, whereby individual feeding behaviors can be recorded. For example, pre-weaned calves diagnosed with BRD drank less milk on the day of clinical examination (47) and on the first day of treatment (53), drank milk slower 4 days prior to the clinical examination (53), and performed fewer unrewarded visits to the milk dispenser 3 days prior to (52), and on the first day of treatment (53). Moreover, net daily energy intake (calculated for each calf by summing daily milk replacer and concentrate intake values) (52) and DMI (55), were reduced in BRD-infected calves in the time prior to the clinical examination, e.g., 3 days in Johnston et al. (52) and 6.8 days in Jackson et al. (55). In calves diagnosed with NCD, daily milk intake and time at water trough dropped 4 days prior to clinical examination (7).

#### Changes in Activity

Changes in activity parameters, such as step counts and lying behaviors, are used to detect sick calves. In calves diagnosed with BRD, for example, step counts (<6 days), lying bouts (<5 days), standing time (<1 day) were reduced (81). In calves diagnosed with NCD, results are inconclusive regarding activity: lying bouts were found to both decrease (<7 days) (7) and increase (<7 days to 3 days) (63), and lying durations were found to both decrease (<6 days to 3 days) (63) and increase (<7 days) (7). Finally, calves with inflamed navels show reduced lying time at day level after arrival at fattening farms compared with healthy calves (56).

#### Changes in Other Parameters

Coughing which is a typical symptom of BRD can be detected using microphones (83). Note that as opposed to activity and feeding behaviors, coughing has so far only been measured at group level. An increased coughing frequency was found to be correlated to BRD occurrence in group-housed calves (51, 65).

Changes in body temperature can be used to detected sick calves before clinical examination. BRD-diagnosed calves showed increases in orbital (eye plus 1 centimeter surrounding the eye) maximum temperature (16) and reticulo-ruminal temperature, e.g., −136 to −12 h (24) and −3.5 days (46) relative to diagnosis. One important methodological consideration with thermometers is that recorded temperatures differ based on the body area that is investigated. For example, skin temperature was consistently 2 to 3°C lower than the rectal temperature (39), while reticulo-rumen temperature was consistently 0.57°C higher than rectal temperature (24). As long as these differences between recorded temperature and body temperature are consistent, this should not affect the detection of temperature increases due to diseases in calves. In calves diagnosed with NCD, the temperature of the side flank and shoulder increased at least seven days prior to diagnosis (7).

Changes in social behaviors were also detected in sick calves. In a modeled disease challenge study (calves were infected with Mannheimia haemolytica), sick calves were found to show decreases in daily social grooming time and daily social lying time (lying within one body length of another calf) (48).

Some other behavioral parameters can be well recorded by sensors, but their potential in early disease detection is yet unknown. These include sucking behavior (38), rumination time (86), and play behavior (93–95). Further research into the link between these parameters and disease is warranted.

As explained above, behavior and physiology change with disease, and these changes can be detected using sensors and algorithms. Theoretically, “behaviors that are less critical for immediate survival and primarily support long-term fitness are most affected by disease” (96), such as play and exploratory behaviors (97). In practice, the type of diseases and the age of the animals also need to be taken into consideration as they might influence behavioral deviations. For example, in parasitized beef steers (aged between 4 and 11 months) and BRD-infected dairy calves, changes in activity (e.g., lying, standing, and step counts) enabled a better disease detection than feeding behaviors such as frequency and duration of feeding and drinking (66) and feed intake (47). In identifying NCD-infected calves and BRD-infected steers, however, feeding behaviors (e.g., the number of unrewarded visits to an automated milk dispenser, DMI, and bunk visit duration) permitted a more accurate detection of disease compared with activities such as lying and standing duration (4, 72). In addition, certain diseases result in behavioral changes that are easier to detect at an earlier stage. NCD-diagnosed calves, for example, displayed earlier and more consistent changes in feeding behaviors compared with BRD-diagnosed calves (53). Further research is hence needed into identifying the best, most sensitive behavioral and physiological parameters that can identify specific diseases or diseased state on a generic level.

### Stage Three: Information Integration–Outcomes of Models

To date, sensor fusion (i.e., two or multiple sensors) was applied in a number of studies (*n* = 9, Figure 3), in which data from accelerometers, thermometers, and AFS are integrated into one model to identify diseases including BRD (64, 72) and NCD (4, 7). Information integration, however, means more than a multiple-sensor tool. First, “integration” does not mean accumulating all the data obtained from different sources. In the design of systems at stage three, redundancy needs to be reduced for a disease-detection model. To reach this, data mining (98), which allows for a more complete understanding of different parameters in relation to disease occurrence, is a prerequisite. Data mining allows for the selection of the key parameters, the variation of which reflects health status with high accuracy. In this way, by reducing the redundancy, the number of sensors used and possibly attached to a calf will be reduced. Second, multiple data sources mean that sensor data are not the only sources of data. Economic insights, for example, were also suggested to be considered for the treatment decisions (24, 57).

Given that many sensors and techniques are already commercially available, it is crucial to choose an appropriate sensor system when recoding certain parameters. For example, the combination of video cameras and sensors (including thermometers, accelerometers, or AFS), although popular for research (*n* = 5), seems impractical for on-farm settings. This might be due to the number of cameras required and the time-consuming process of analyzing the video footage. However, a recent study show that artificial intelligence is able to identify the physiology and behavior of animals using video footages with high accuracy (90), allowing for less labor.

Therefore, “information integration” means selecting as few meaningful parameters indicative of diseases as possible when developing models (thereby avoiding redundancy). The integrated systems will give an alert when the current status of a calf deviates from its earlier patterns, i.e., being sick. Ideally, models at this stage include a minimum number of sensors per animal, which is advantageous in terms of costs and maintenance labor but also in maintaining the integrity and freedom of movement of calves.

### Stage Four: Decision Support–Automation

At stage four, decision support means that the integrated system can identify which disease is occurring based on the developed model. Farmers can refer to the decision made by the system as support. To the author's knowledge, no such systems are available for early disease detection in calves. An example of a stage four system in dairy cows is estrous detection and automatic identification of the best way to inseminate the cow (99). In the situation of early disease detection in calves, however, so far only alerts are available.

For the future, automation is crucial–a decision-support system with an easy-to-operate user interface is what farmers need for an easy identification of sick calves. Current models may give some form of alert, yet cannot give automatic decision support. Another important characteristic of such systems is the possibility for the farmers to enter feedback (e.g., whether the identified individual was truly ill with the suspected disease and whether the treatment was efficient) so that the system can continuously learn and adapt to the specific farming conditions. Knight (98) suggested a business model that bridges information integration and decision support. In the provided business model, farmers are buying a service from service providers. A service provider purchases the technologies from different developers, and provides the service of installation, maintenance, data collection, and data integration, thereby providing decision support to the farmers. However, at the same time we believe that technology should not replace but, rather, support management decisions made by the farmer.

### Introducing “Resilience” Theory

Following the above four-stage approach, the decision-support system is regarded as a screening tool that aims to detect diseases at an early stage and provides a short list of “positive cases” of animals that have a sufficiently high chance of being prone to develop the targeted disease. Hence the farmer and the care taker is provided a window of opportunity to check for the clinical status. Additionally some preventive measures can be taken or a predefined health protocol can be applied. A negative outcome of a decision-support system predicts the likelihood of a clinically healthy animal of becoming diseased in the near future, i.e., its predisposition to diseases then is generally speaking a balance between environmental infection pressure and its immune system functioning. In a paper discussing sensor technologies in dairy farming, Knight argued that “the focus is on improving overall husbandry, rather than “solving” specific disease problems” (98). The same focus should apply to the dairy and veal industry as well. We therefore introduce “resilience” theory, through which the developed system might be able to quantify the resilience of individual animals, thereby identifying animals in a low-resilience state. This potentially allows for early intervention in the husbandry system, whereby the environment or management are specified in such a way that low-resilient individuals and the herd as a whole can maintain relatively healthy states.

Resilience in farm animals has been defined as “the capacity of the animal to be minimally affected by a disturbance or to rapidly return to the physiological, behavioral, cognitive, health, affective and production states that pertained before exposure to a disturbance” (100). Calves falling sick can be equaled to a complex system transiting from one stable state (healthy) to another (unhealthy), with the return to the original state being more difficult than the simple cancellation of factors that caused the change in state. Such shifts in complex systems have been termed “critical transitions” or “tipping points” (101). When such complex systems are close to tipping points, the recovery rate of that system from small perturbations becomes very slow, and this is known as “critical slowing down” (CSD) (102). For example, a cow showing “CSD” before parturition, in this case by using an accelerometer to assess activity (e.g., low average eating time, a disturbed circadian rhythm, and variance in ear temperature), is likely to develop periparturient disorders (103). CSD, which can be revealed through dynamic aspects of sensor data, is here seen as an increase in variance in the activity data, hence a loss of regularity. CSD, therefore, reflects a loss of resilience (101, 102). In still clinically healthy individuals, CSD reflects the animal's vulnerability to pathogens prior to the disease, and hence reflects a state of low resilience. Identifying CSD in sensor data patterns of “low-resilient” individual animals, would enable, for example, timely change of the environment of this animal in an attempt to increase its resilience (e.g., by removal of stressors, or the improvement of nutrition, etc.).

Current sensor tools focus on detecting the early stages of disease, while sensor technology already allows us to analyze the dynamics of physiology and behavior with high accuracy. Advanced analytical tools can estimate resilience status from the micro-recoveries in the data flow (104). These tools may eventually also be applicable to advanced and innovative calf health management systems.

## Conclusions

This review summarized the literature on sensor systems so far studied in the context of health monitoring in calves between 2009 and 2021, and revealed the current phase of development by categorizing each study based on a four-stage system (sensor technology, data interpretation, information integration, decision support). Our literature search demonstrated that most studies up to now are at stage one (sensor technique) or stage two (data interpretation), and a few studies are at the beginning of stage three (information integration). Accelerometers, IRT cameras, microphones, and 3-D cameras can be accurate in measuring behavioral and physiological parameters in calves (at stage one). Deviations in behaviors (e.g., feeding, lying, and social behaviors), activity, and body temperature can be detected prior to the clinical examination (at stage two and three), and are promising for developing algorithms. To develop a health detection model with a minimal number of sensors, it is crucial to select appropriate sensor systems that can record the most relevant parameters that show clear changes in response to diseases in calves. Clear gaps in research include stage three (information integration) and stage four (decision support) systems, as well as forecasting methods via the identification of low-resilience animals.

## Data Availability Statement

The original contributions presented in the study are included in the article/Supplementary Material, further inquiries can be directed to the corresponding authors.

## Author Contributions

DS wrote the review (main body) and LW, PT and KR contributed during the discussion and reviewed the preliminary versions of the manuscript. All authors contributed to the article and approved the submitted version.

## Funding

This study was financially supported by Stichting Brancheorganisatie Kalversector (SBK), the Dutch Ministry of Agriculture, Nature and Food Quality.

## Conflict of Interest

The authors declare that the research was conducted in the absence of any commercial or financial relationships that could be construed as a potential conflict of interest.

## Publisher's Note

All claims expressed in this article are solely those of the authors and do not necessarily represent those of their affiliated organizations, or those of the publisher, the editors and the reviewers. Any product that may be evaluated in this article, or claim that may be made by its manufacturer, is not guaranteed or endorsed by the publisher.
