# Supplementary material for: A Systematic Review of Automatic Health Monitoring in Calves: Glimpsing the Future From Current Practice
Source: Front Vet Sci. 2021 Nov 26;8:761468. doi: 10.3389/fvets.2021.761468 (PMC8662565; doi:10.3389/fvets.2021.761468)
Supplement: Supplementary file 1 [file Table_1.DOCX]

| **No.** | **Reference** | **Equipment and technique** | **Objectives** | **Indicators** | **Gold standards** | **Animal** | **Sample size (number of animals)** | **Assessment** | | | | | **Level^a^** |
| --- | --- | --- | --- | --- | --- | --- | --- | --- | --- | --- | --- | --- | --- |
|  |  |  |  |  |  |  |  | **Sensitivity** | **Specificity** | **Accuracy** | **Precision** | **Other** |  |
| 1 | Borderas et al. (2009) | Automatic feeding stations (AFS, manufacturer not mentioned) | Morbidity (BRD and NCD) | Feeding behavior | Clinical examination | Pre-weaned dairy calves | Exp. 1 (low milk ration): 26, Exp. 2 (high milk ration): 38 | - | - | - | - | - | 2 |
| 2 | Knauer et al. (2018) | AFS (Förster-Technik GmbH, Engen, Germany) | Morbidity (including BRD and NCD) | Feeding behavior | Clinical examination, calf enrollment, treatment record, morbidity and mortality data | Pre-weaned dairy calves | 176 pairs (176 treated, 176 healthy) | * | * | - | - | * (positive predictive value, negative predictive value) | 2 |
| 3 | Szyszka et al. (2012) | Video camera, accelerometers (Icetag, IceRobotics, Roslin, UK) | Morbidity | Feeding and water drinking behavior; activity (number of steps) and posture (lying time, standing time) | BW; biochemical analysis from blood and fecal samples; rectal temperature | Beef bulls (aged between 4 and 11 months) | 17 | - | - | - | - | - | 3 |
| 4 | Oliveira et al. (2018)^b^ | AFS (feed bin and water bin) (Intergado Ltd, Contagem, Minas Gerais, Brazil) | Behavior observation | Feeding and water drinking behavior, water and feed intake | Video recording (feeding and drinking behaviors) and manual weighing (water and feed intake) | Holstein–Gyr crossbred heifers | 35 (4-week) | * | * | - | - | * [r(r^2^)] | 1 |
| 5 | Robért et al. (2011) | Accelerometer (Accelerometers consisted of a triaxial, capacitance-type, ± 10-g integrated circuit and recorded data at 100 Hz) | Behavior observation | Walking, standing, lying behaviors | Clinical examination | Beef steers | 25 (20 day) | - | - | - | - | - | 1 |
| 6 | Roland et al. (2018)^c^ | Accelerometer (Smartbow ear tag, Smartbow GmbH, Weibern, Austria) | Behavior observation | Milk drinking behavior | Behavior recorded by infrared video cameras | Pre-weaned dairy calves (bucket-fed) | 3 (5 days of observation per calf) | * | * | * | * | - | 1 |
| 7 | Swartz et al. (2016) | Accelerometer (AfiTag II, Afimilk Ltd., Kib- butz Afikim, Israel) | Behavior observation | Lying behavior, step activity | Lying and step behavior recorded by video cameras | Pre-weaned female dairy calves | 5 (10-day sensor recording, 7-hour video recordings) | - | - | - | - | * (r) | 1 |
| 8 | Swartz et al. (2017) | Accelerometer (AfiTag II, Afimilk Ltd., Kibbutz Afikim, Israel), AFS (FA Förster-Technik GmbH, Engen, Germany) | BRD | Step counts, lying and feeding behaviors | Clinical examination | Pre-weaned calves | 30 | - | - | - | - | - | 2 |
| 9 | de Passillé et al. (2010) | Accelerometer (Hobo Pendant G Acceleration Data Logger, Onset Computer Corporation, Pocasset, MA, USA) | Behavior observation | Step counts, gait classification (galloping, trotting, walking) | Behavior classification based on video recording | Female dairy calves | 7 | - | - | - | - | * (r) | 1 |
| 10 | Ferrari et al. (2010) | Microphone (Monacor ECM 3005), audio labelling tool | BRD | Cough sounds | ~~-~~ | Weaned dairy calves | 28 (5-day data collection) | - | - | - | - | - | 1 |
| 11 | Vandermeulen et al. (2016) | Pig cough monitor system (SoundTalks, Leuven, Belgium) | BRD | Cough sounds | Clinical examination; blood neutrophils number from blood sampling | Male dairy calves | 62 (continuously recorded over a 3-month period) | * | * | - | * |  | 2 |
| 12 | Carpentier et al. (2018) | Sound acquisition system (SoundTalks NV, Belgium) | BRD | Cough sounds | Blood analysis | Bull calves | 139 (445 min of data) | * | - | * | * | - | 2 |
| 13 | Timsit et al. (2011) | Reticulo-rumen temperature boluses (Thermobolus, Medria SAS) | BRD | Reticulo-rumen hyperthermia (RH) | Clinical examination; serum haptoglobin concentration | Young bulls (fattening period) | 24 (first 40 days after arrival) | - | - | - | - | * (r) | 2 |
| 14 | Voss et al. (2016) | Reticulo-rumen temperature boluses (Medria, Châteaubourg, France) | BRD | Reticulo-ruminal (ReRu) temperature | Clinical examination | Pre-weaned male calves | 150 (8-week) | * | * | - | - | * (r) | 2 |
| 15 | Schaefer et al. (2012) | Infrared thermal station (around animal's water station, designed and developed at the Lacombe Research Centre, Alberta, Canada) | BRD | Orbital maximum temperature | Clinical examinationand biochemical analysis | Receiver calves | 65 (3-week) | * | * | - | - | * Positive predictive value, negative predictive value) | 2 |
| 16 | Nogami et al. (2013) | Thermometer integrated into an accelerometer (attach to the base of the tail) | BRD | Body surface temperature (tail temperature); activity | Rectal temperature | Calf | 1 (16 days of continuous measurement) | - | - | - | - | * (r^2^) | 1 |
| 17 | Toaff-Rosenstein et al. (2017) | RFID | Behavior observation | Grooming behavior: brush proximity | Brush contact duration obtained by video observation | Angus and Hereford heifers | 16 (6 hour per heifer) | * | * | - | - | - | 1 |
| 18 | Hixson et al. (2018) | Video cameras, accelerometer (Hobo Pendant G Acceleration Data Logger, Onset Computer Corp., Pocasset, MA, USA) | BRD | Lying behavior; feeding behavior | Clinical examination | Bull calves | 12 BRD-challenged calves, 12 as control (loggers were attached 12 days before inoculation and set to record for 7 days following inoculation) | - | - | - | - | - | 3 |
| 19 | Sutherland et al. (2018) | (AFS; from A&D Reid, Temuka, New Zealand), accelerometers (Hobo Pendant G Acceleration Data Logger, 64k, Onset Computer Corporation, Pocasset, MA, USA) | NCD | Feeding and lying behavior | Clinical examination; Biochemical analysis; fecal sampling; BW | Dairy calves | 36 (18 pairs) | - | - | - | - | - | 3 |
| 20 | Studds et al. (2018) | Accelerometers (Hobo Pendant G Acceleration Data Logger, Onset Computer Corporation, Pocasset, MA, USA) | Diarrhea and navel inflammation | Lying behavior | Clinical parameters by health exams: rectal temperature, navel score and fecal score | Male neonatal veal calves | 125 (first 2 weeks after arrival) | - | - | - | - | - | 2 |
| 21 | Kour et al. (2018) | Accelerometer (USB AccelerometerX16-4, weighing 48 g, Gulf Coast Data Concepts, LLC, Waveland, MS, USA) | Behavior observation | Sucking behavior | Sucking behavior from visually recorded behavioral data | Cow-calf pairs (Belmont red cattle) | Exp. 1 (model development): 2 (2-day period); Exp. 2: 23 (10-day period); Exp. 3: 21 (visually observed for 6 hours for 3 consecutive days) | - | - | * | - | - | 1 |
| 22 | Oliveira et al. (2018)^d^ | AFS (including automatic water bin system) | Tick-borne diseases | Feeding behavior | Blood analysis; clinical examination | Heifer calves | 35 (12 calves detected with tick-borne disease were included for final analysis, 4-week period) | - | - | - | - | - | 2 |
| 23 | Johnston et al. (2016) | AFS (Vario Powder (Förster-Technik GmbH, Engen, Germany) and concentrate (KFA3-MA3; Förster- Technik GmbH, Engen, Germany) | BRD | Feeding behavior | Clinical examination and biochemical parameters from blood sample | Pre-weaned bull calves | 64 | - | - | - | - | - | 2 |
| 24 | Shane et al. (2018) | Real-time location system (accelerometer) (Smartbow ear tags, Smartbow GmbH, Weibern, Austria) | BRD | Social network patterns: contact between calves | A 4-point clinical illness scoring chart | Weaned beef steers | 70 (first 28 days after feedlot entry) | * | * | * | - | * | 2 |
| 25 | Jackson et al. (2016) | AFS (GrowSafe Systems Ltd., Airdrie, AB, Canada) | BRD | Daily DMI and feeding behaviors | Clinical parameters of BRD based on clinical signs of illness and BW | Growing bulls | 231 (70-day) | - | - | - | - | - | 2 |
| 26 | Roland et al. (2018) | Accelerometer (Smartbow eartags, Smartbow GmbH, Weibern, Austria) | Behavior observation | Postures, activities | Activities and behaviors observed from infrared video recording; visual observations | Pre-weaned dairy calves | 15 (38 hours of acceleration data and video observation) | * | * | * | * | - | 1 |
| 27 | Nir et al. (2018) | 3D-camera (Microsoft Kinect v2 sensor) | Growth (animal dimension) | Body mass, hip height, withers height | Manual measurement | Dairy heifers | 107 | - | - | - | - | * (r^2^) | 1 |
| 28 | Pezzuolo et al. (2018) | 3D-camera (Microsoft Kinect™ v1 RGB-depth camera) | Growth | Body mass | Manual measurement | Calves and cows | 20 | - | - | - | - | * (r^2^) | 1 |
| 29 | Burfeind et al. (2011) | Microphone (Hi-Tag rumination-monitoring system (SCR Engineers Ltd., Netanya, Israel) | Behavior observation | Rumination time (RT) | RT by visual observation | Heifer and calves | 44 | - | - | - | - | * (r) | 1 |
| 30 | Rodrigues et al. (2019) | Hr-Tag rumination monitoring system (SCR Engineers Ltd., Netanya, Israel) | Behavior observation | RT | RT by visual observation | Weaning calves | 32 | - | - | - | - | * (r) | 1 |
| 31 | Lopreiato et al. (2018) | Hr-Tag rumination-monitoring system (Hr-Tag; SCR Engineers Ltd., Netanya, Israel) | Behavior observation | RT | - | Pre-weaned calves | 9 | - | - | - | - | - | 1 |
| 32 | Rodriguez-Baena et al. (2020) | Accelerometer (Hobo Pendant G Data  Logger [UA-004-64]) (as collar) | Behavior observation (pattern recognition) | Eating (head in hopper),  drinking (head in water trough), chewing, walking,  positive social interaction, self-grooming,  other kind of activity, Inactive | Visual annotations from animals’ behavior | Weaner calves (aged between 6 and 8 months) | 20 (4 weeks) | * | - | * | * | - | 1 |
| 33 | Bonk et al. (2013) | Accelerometer (Hobo Pendant G Acceleration Data Logger, Onset Computer Corporation, Pocasset, MA, USA) | Behavior observation | Lying behavior | Lying behavior from scan sampling (by camera) | Dairy calves (Exp.1 = 8 (aged 21.5 ± 14.5 days), Exp.2 = 19 (aged 29.4 ± 4.6 days) | Exp. 1: 8 (37 2-hour observations for the individual housed calves, 10 × 2-hour observations for the 4 group-housed calves), Exp. 2: 19 (24-hour recording period) | * | * | - | - | * (r) | 1 |
| 34 | Trénel et al. (2009) | Accelerometer (IceTag; IceRobotics,  Edinburgh, UK) | Behavior observation | Lying, standing, and activity | Lying, standing, and moving behaviors recorded by video recordings | Dairy calves | 9 | * | * | - | - | * (Se + Sp) | 1 |
| 35 | Finney et al. (2018) | Accelerometer (tri-axial 4 Hz IceQube accelerometer, IceRobotics, Edinburgh, Scotland) | Behavior observation | Lying duration | Daily lying durations by retrospective analysis of video recordings | Weaning dairy calves | 13 (96 continuous hours) | - | - | - | - | - | 1 |
| 36 | Moya et al. (2015) | AFS (GrowSafe Systems, Airdrie, AB, Canada) | Morbidity (BRD) | Feeding behavior | Clinical parameters, carcass information, lung lesions at slaughter | Heifers | Model dataset: 384 (225-day feeding period); validation dataset: 384 (142-day feeding period) | * | * | * | - | - | 2 |
| 37 | Knauer et al. (2017) | AFS (Kalb Manager, Förster-Technik GmbH, Engen, Germany) | Morbidity | Feeding behaviors | Clinical parameters, chemical parameters in blood, calf morbidity and mortality, and enrollment data | Pre-weaned dairy calves | 352 (matched pair analysis), enrolled calves: 1,052 (43,607 calf days over 9 months) | - | - | - | - | - | 2 |
| 38 | Kayser et al. (2019) | AFS | BRD | Feeding behaviors | Clinical parameters for gastroenteric and respiratory affections | Growing bulls | 231 (70-day) | * | * | * | - | - | 2 |
| 39 | Scoley et al. (2019) | IRT camera (E8, FLIR Systems UK, Kent, UK) | Temperature assessment | Left and right eye (defined as the whole eye + 1 cm margin), rectal area (defined as the anus +1.5 cm margin) | Rectal temperature | Calves aged under 12 weeks | Experiment 1: 16, experiment 2; 12, experiment 3: 205 | - | - | - | - | r | 1 |
| 40 | Bell et al. (2020) | IRT camera  (SC620 FLIR Comp, Boston, MA) | Pyrexia detection | Thermal imaging temperature (around the medial canthus of the eye) | Rectal temperature | Pre-weaned calves (aged from 7 to 40 days) | 125 (100 pure-bred Holstein male, 25 pure-bred Holstein female calves) | - | - | - | - | r | 1 |
| 41 | Toaff-Rosenstein et al. (2016) | Temperature logger (TidbiT v2 number UTBI-001, Onset Computer Corp., Pocasset, MA), video cameras | BRD | Video recording (feeding time, self-licking, brush contact),  rectal temperature | Clinical score and necropsy | Steers | 20 (4 treatments × 5 steers per treatment) | - | - | - | - | - | 3 |
| 42 | Toaff-Rosenstein and Tucker (2018) | Temperature logger (TidbiT v2 # UTBI-001, Onset Corp., Pocasset, MA, USA), video cameras | BRD | Feeding time, grooming behavior (brush use), rectal temperature | Clinical signs based on clinical parameters, other systemic or critical conditions | Beef heifers | 67 (monitored for 12 days, started 2 days after arrival) | - | - | - | - | - | 3 |
| 43 | Hanzlicek et al. (2010) | Pedometers (NL-800 Activity Monitor, New Lifestyles, Lee’s Summit, MO, USA), accelerometers (GP 1Progammable Accelerometer, Sensr, Elkader, IA, USA) | BRD | Step numbers (by pedometers); lying, standing, and walking (by accelerometers) | Physical examination, clinical illness score, and degree of activity; arterial blood gas measurements, serum biochemical analyses, and complete blood counts | Post weaned calves (beef steers) | 14 | - | - | - | - | - | 3 |
| 44 | Wolfger et al. (2015)^e^ | AFS (Growsafe Systems Ltd., Airdrie, AB, Canada) | BRD | Feeding behavior | Clinical parameters from clinical assessment, biochemical parameter from blood sample | Beef steers | 213 (35-day) |  |  |  |  | - | 2 |
| 45 | Wolfger et al. (2015)^f^ | Accelerometer (SensOor, Agis Automatisering BV, Harmelen, The Netherlands) | Behavior observation | Feeding, rumination, active, resting | Live observation from video recording | Yearling Steers | 18 (13-day) | * | * | - | - | * (concordance correlation) | 1 |
| 46 | Guo et al. (2020) | 2-D camera (DS-2CD4012, Hikvision, Hangzhou, China) | Behavior observation | Scene-interactive behaviors: pen entering, pen leaving, staying (standing or laying static behavior), turning, feeding and drinking | Video respective analysis | Dairy calf (aged 2 months) | 1 | * | - | - | - | - | 1 |
| 47 | Lowe et al. (2020) | Infrared camera (FLIR Systems AB, Danderyd, Sweden) integrated into each calf feeder | Temperature assessment | Eye and cheek temperatures | Manual image analysis | Heifer calves (aged from 2 to 24 days) | 120 | - | - | - | - | R^2^ | 1 |
| 48 | Kayser et al. (2020) | AFS (GrowSafe Systems Ltd., Airdrie, AB, Canada), accelerometers (Feed Phone system, Medria, Châteauborg, France), radiofrequency biothermal boluses (ThermoBolus, Medria, Châteauborg, France) | BRD | DMI, feeding behaviors, activities | Clinical score, rumen temperature, hemogram | Angus crossbred steers (initial BW = 386 ± 25 kg) | 36 (56 days: data were collected for 28 days prior to and following inoculation) | * | * | * | - | - | 3 |
| 49 | Lowe et al. (2019)^g^ | Infrared camera (T650sc, FLIR systems AB, Danderyd, Sweden) | Respiratory rate (breaths/min) assessment | Respiratory rate (observing thermal fluctuations around the nostrils during inhalations and exhalations from infrared recordings) | Flank movements from video recordings | Calves | 5 | - | - | - | - | R^2^ | 1 |
| 50 | Lowe et al. (2019)^h^ | Automated calf feeder, hand-held IRT camera (ThermaCAM S60; FLIR Systems AB, Danderyd, Sweden), accelerometers (Hobo Pendant G data loggers, Onset Computer Corporation, Pocasset, MA, USA), video cameras ((HC-V270, Panasonic, Osaka, Japan) | NCD | Feeding behaviors, lying behaviors, drinking behavior, infrared thermography temperatures(eye, cheek, back, shoulder, and side) | Clinical examination, fecal sample analysis, respiratory rate | Calves | 43 | - | - | - | - | - | 3 |
| 51 | Pillen et al. (2016) | Accelerometers (lceQube, lceRobotics, Ltd., Midlothian, Scotland, UK) | BRD | Standing time, step count, lying bouts, motion index | Clinical assessment, depression score | Beef calves | 364 (56-day) | - | - | - | - | - | 2 |
| 52 | Carslake et al. (2021) | Collor-mounted sensors comprise an accelerometer and gyroscope (SparkFun 9DoF Razor IMU M0, SparkFun Electronics, Niwot, CO, USA) | Behavior observation | Locomotor play, self-grooming, ruminating, non-nutritive suckling, nutritive suckling, active lying, non-active lying | Video respective analysis | Pre-weaned dairy calves | 13 (12-day: 2 days were used as pilot, 10 days for data collection) | * | * | * | * | F-score, Cohen’s Kappa | 1 |
| 53 | Swartz et al. (2020) | Accelerometers (AfiTag II, Afimilk Ltd.,  Kibbutz Afikim, Israel) | NCD | Step activity, lying behavior | Clinical examination | Pre-weaned calves | 30 | - | - | - | - | - | 2 |
| 54 | Duthie et al. (2021) | Automatic calf feeder (Holm and Laue version 100, Holm and Laue, Westerrönfeld, Germany), accelerometers (3-axis accelerometer: Axivity AX3, Axivity Ltd., Newcastle upon Tyne, UK) | BRD | Feeding behavior, activity | Clinical examination | Pre-weaned dairy calves | 100 | - | - | - | - | - | 3 |

^a^ Level 1: sensor technique, level 2: data interpretation, level 3: information integration, level 4: decision support.

^b^ Oliveira BR, Ribas MN, Machado FS, J. Lima AM, Cavalcanti LFL, Chizzotti ML, et al. Validation of a System for Monitoring Individual Feeding and Drinking Behaviour and Intake in Young Cattle. Animal (2018) 12:634–639. doi:10.1017/S1751731117002002.

^c^ Roland L, Lidauer L, Sattlecker G, Kickinger F, Auer W, Sturm V, et al. Monitoring Drinking Behavior in Bucket-Fed Dairy Calves Using an Ear-Attached Tri-Axial Accelerometer: A Pilot Study. Comput Electron Agric (2018) 145:298–301. doi:10.1016/j.compag.2018.01.008.

^d^ Oliveira Júnior BR, Silper BF, Ribas MN, Machado FS, Lima JAM, Cavalcanti LFL, et al. Short Communication: Tick-borne Disease is Associated With Changes in Feeding Behavior in Automatically Fed Weaned Dairy Calves. J Dairy Sci (2018) 101:11256–61. doi: 10.3168/jds.2018-14637

^e^ Reference 61.

^f^ Reference 73.

^g^ Reference 88.

^h^ Reference 7.
